# Nitrogen-doped mesoporous SiC materials with catalytically active cobalt nanoparticles for the efficient and selective hydrogenation of nitroarenes

**DOI:** 10.1038/s41598-018-20976-z

**Published:** 2018-02-07

**Authors:** Mirco Eckardt, Muhammad Zaheer, Rhett Kempe

**Affiliations:** 1 Lehrstuhl fuer Anorganische Chemie II-Katalysatordesign, Universitaet Bayreth, Universitaetstr, 30, 95447 Bayreuth, Germany; 2 Department of Chemistry and Chemical Engineering, SBA School of Science and Engineering, Lahore University of Management Sciences (LUMS), 54792 Lahore, Pakistan

## Abstract

Mesoporous nitrogen-doped silicon carbide catalysts with integrated cobalt nanoparticles (Co@N-SiC) were synthesized by the thermal decomposition of a microphase-separated block copolymer of polycarbosilane and polyethylene. The catalysts are highly active, reusable and offer selective hydrogenation of the nitro group in the presence of hydrogenation-sensitive functional groups.

## Introduction

Intensive efforts have been devoted recently to designing novel catalysts with enhanced catalytic properties utilizing earth-abundant transition metals^1–3^. Cobalt, among other metals, has attracted many researchers and numerous homogeneous^4–6^ and heterogeneous cobalt catalysts^7–18^ have been developed for various catalytic transformations. Beller and co-workers^19^, for instance, developed a cobalt-oxide@N-carbon catalyst by the thermal decomposition of a specific cobalt complex for the selective hydrogenation of nitroarenes. This is an industrially important reaction, as aniline and its derivatives find applications in the synthesis of pharmaceutical, dyes, polymers, agrichemicals and other fine chemicals^20,21^. Moreover reduction of nitroarenes has served as a benchmark reaction to test the activity of nanoparticles (NPs)^22^. Thus, the development of new catalysts with important features, such as economy, activity, selectivity and reusability, is highly relevant^23^. In the past various catalysts based on Au^24^, Ag^25^, Ni^26^, Fe^27^ and Cu^28^ has been designed for the reduction of nitroarenes. Since the pioneer work of Beller, a variety of Co and especially Co@N-carbon catalysts have been developed^29–43^. Recent studies have shown that the stability of Co-N-C bond plays a vital role in the temperature-dependent formation of cobalt single atoms to NPs^44^. N-functions of the support are crucial not only for the stabilization of metal NPs, but also for the dispersion of the catalyst in polar solvents used for the catalysis^45^. Moreover, an increase in the activity with an increase in nitrogen content of the catalyst was observed^46^. Silicon carbide (SiC) and silicon carbonitride (SiCN), being refractory materials, could extend the application profile of cobalt-based solid catalysts if used as a catalyst support. We have demonstrated the catalytic potential of metal@SiCN materials for various transformations^47–54^, including a cobalt-based catalyst for the reduction of nitroarenes and the direct synthesis of imines and benzimidazoles from nitroarenes and aldehydes^55^. In addition, we introduced an approach for the one-step synthesis of porous SiC materials^56^.

Here, we report the development of a N-doped mesoporous SiC cobalt nanocomposite material (Co@N-SiC) which affords remarkable activity, selectivity and reuse in the hydrogenation of nitroarenes.

## Results and Discussions

**Figure 1:**
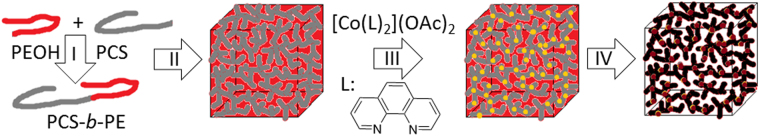
Synthesis of mesoporous Co@N-SiC ceramics; (I) block copolymer formation; (II) microphase separation; (III) metallation with a Co complex [Co(L_2_)(OAC)_2_] where L: phenanthroline, OAc: acetate; (IV) pyrolysis.

The synthesis of Co@N-SiC is summarized in Fig. 1, while details are provided in the supporting information (SI). A commercial polycarbosilane (PCS) was reacted with a hydroxyl-terminated polyethylene (PEOH)^57^ to synthesize a PCS-block-PE (PCS-*b*-PE) polymer via dehydrocoupling of Si-H and O-H bonds^56^. A cobalt complex ([Co(phen)_2_](OAc)_2_ where phen: phenanthroline and OAc: acetate) was added to this block copolymer and the melt was slowly cooled to room temperature to achieve nanostructuring. The PEOH and the cobalt complex decompose upon pyrolysis (under nitrogen) (see TGA in Figure S1), leaving behind an amorphous SiC material with integrated cobalt NPs. The formation of SiC ceramics was confirmed by FT-IR analysis (see Fig. S2 in SI). The absence of characteristic bands at 2852 and 2926 cm^−1^ (C-H stretching vibration) and those at 2200 cm^−1^ (Si-H) confirmed the removal of the organic block and the formation of SiC material.

**Figure 2:**
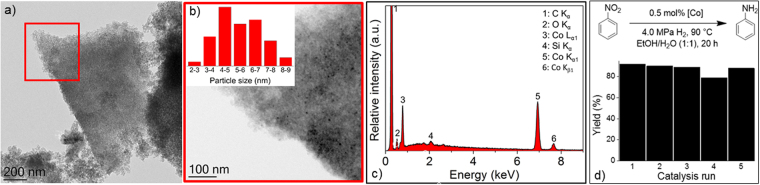
Transmission electron microscopy images of Co@N-SiC catalysts (**a** and **b**), magnified view of selected area from (**a**) is shown in (**b**), where the inset shows the size distribution of Co NPs (encircled). The EDX of the catalyst is presented in (**c**). Reusability of the catalyst is presented in (**d**).

Co@N-SiC was investigated by transmission electron microscopy. A piece of the catalyst, as shown in Fig. 2a, indicates a structured, but not highly ordered material. A magnified view of the region selected (red square in Fig. 2a) shows metallic NPs (Fig. 2b) with an average size of 5 nm. Elemental composition of the material was investigated by energy-dispersive X-ray analysis and the results are presented in Fig. 2c. Characteristic peaks corresponding to cobalt can be seen along with those of carbon and some oxygen. The formation of graphitic carbon, as reported earlier^56^, is likely from the decomposition of the organic block. A small amount of oxygen could arise from the PEOH block. Presence of nitrogen (2.5 wt%) in Co@N-SiC material was confirmed through elemental analysis, which is an indication that nitrogen has been successfully incorporated in the material. It would be worth mentioning at this stage that pyrolysis of PCS only provides a non-porous SiC material with no nitrogen doping.

**Figure 3:**
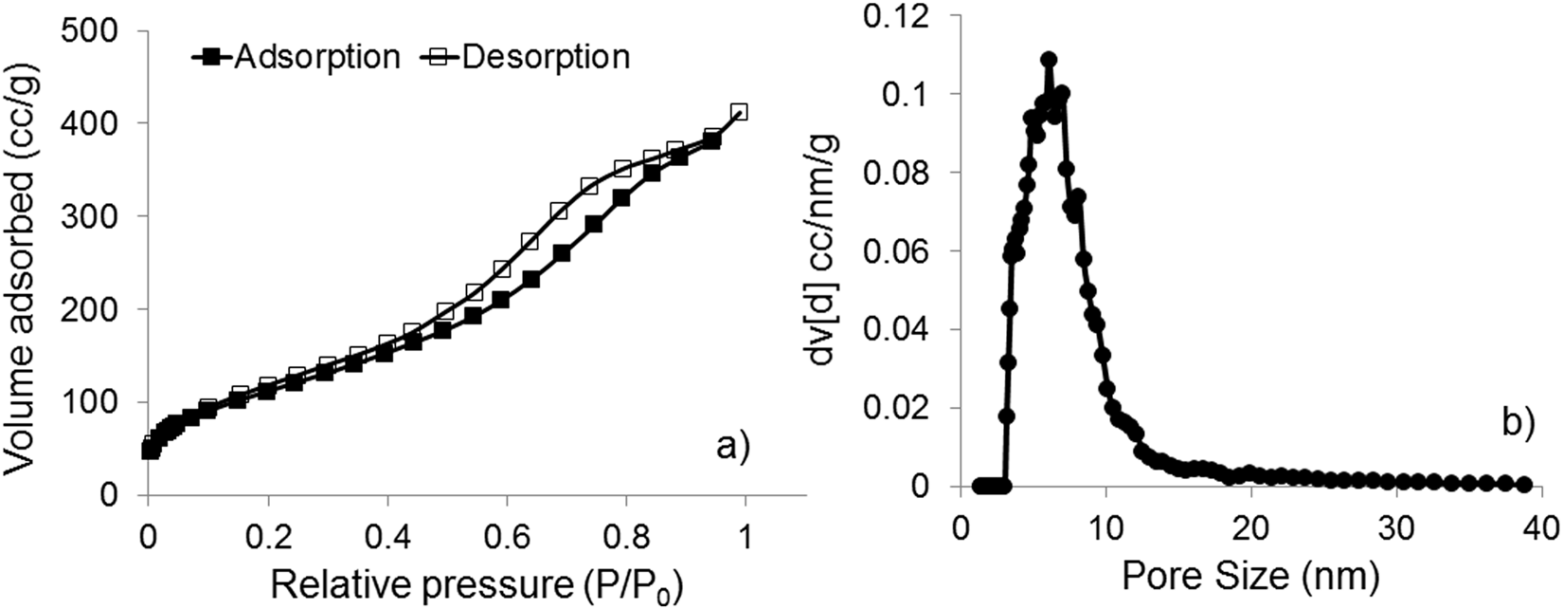
Nitrogen adsorption-desorption isotherm (**a**) and pore size distribution (**b**) of synthesized catalyst.

The presence of permanent porosity in the material was confirmed by nitrogen physisorption. The catalyst showed a high specific surface area (400 m^2^/g) and rather widely distributed mesopores (about 4–15 nm, see Fig. 3b). A hysteresis and closure of the loop near 0.4 P/P_0_ is typical of mesoporous materials in an adsorption-desorption isotherm (Fig. 3a). Although t-plot method^58^ suggested almost no contribution of micropores to the total surface area, the presence of micropores, however could not be ruled out completely because of the significant adsorption of nitrogen at relative pressure values <0.05.

Catalytic activity of the material for the hydrogenation of nitroarenes with molecular hydrogen was investigated without using any catalyst promoter or a base. Firstly, nitrobenzene was used as a substrate and the effect of various parameters, for example, solvent, temperature, reaction time and catalyst loading, was investigated. The highest yield of aniline (90%) during solvent screening (Table T1 in SI) was obtained in water (entry 1) and water ethanol mixture (entry 2), whereas THF was found to be the least effective (entry 3). However, activity of the catalyst decreased in pure ethanol and only 42% of nitrobenzene was hydrogenated under screening conditions. Although the highest activity of the catalyst was found in water, a mixture of ethanol and water was used for further studies due to the insolubility of many of the tested compounds in pure water. The other reaction parameters, such as temperature, time and hydrogen pressure, were also varied and optimized. A complete conversion of nitrobenzene to aniline was achieved under optimized reaction conditions [0.5 mmol nitrobenzene, 90 °C, 4.0 MPa H_2_, 0.5 mol% catalyst metal, 4 mL solvent (EtOH and water 1:1), 20 h]. We tried different ligands (Table T2 in SI) for the synthesis of the Co@N-SiC catalysts and only the complex with phen ligand (entry 3) showed catalytic activity. This observation could be attributed to the ability of phen ligand to retain higher nitrogen content in the catalyst as compared to other ligands tested^46,59^. The catalyst can be used in several successive runs without losing any activity, as can be seen in Fig. 2d. From this, it can be concluded that the cobalt particles are well embedded in the support and that N-functions of the support may play a crucial role regarding the strong catalyst support interactions. A small reduction in catalytic activity was noted only in the fourth cycle, which falls under the GC error. In the last step, selective hydrogenation of the nitro group in the presence of other functional groups was studied and the results are presented in Table 1. Selective hydrogenation of the nitro group without any dehalogenation was observed in the case of nitroarenes with chloro (entries 2–4), bromo (entries 5–7), fluoro (entry 9) and iodo (entry 8) groups. C-C double bond (entry 14), carbonyl group (entry 13) and nitrile (entry 10) functional groups, usually highly sensitive towards hydrogenation, were also not hydrogenated. Sterically demanding arenes, such as 5-nitroisoquinoline (entry 17), were also converted in high yields. The yields were above 95% for all reactions, except for the substrates with sterically demanding substituents, such as bromine (entry 5), phenyl (entry 16), amine (entry 18) or primary alcohol (entry 11) at the ortho position. A dinitro compound was also hydrogenated upon doubling of the catalyst amount (entry 15).

**Table 1:** Hydrogenation of various nitroarenes using the Co@N-SiC catalyst^[a]^.

Entry	Product (Yield)	Entry	Product (Yield)	Entry	Product (Yield)
1		7		13	
2		8		14	
3		9		15	
4		10		16	
5		11		17	
6		12		18	

[a] Reaction conditions: 0.5 mmol nitroarene, 100 °C, 4.0 MPa H_2_, 2 mol% catalyst (0.59 mg Co, 0.01 mmol, 22 mg) [b] 1 mol% of catalyst (0.30 mg, 0.005 mmol of Co, 11 mg); [c] 90 °C, 100 μL H_2_O, 4 mL EtOH; [d] 100 μL H_2_O, 4 mL EtOH; [e] 4 mol% catalyst (1.18 mg Co, 0.02 mmol, 44 mg). *Hydrogenation took place here. Turnover numbers (TON) are provided in parenthesis.

## Conclusions

One-pot synthesis of a nitrogen-doped SiC material containing cobalt NPs (Co@N-SiC) is feasible via the formation, microphase separation and pyrolysis of a polycarbosilane-block-polyethylene polymer modified with a cobalt phenanthroline complex. The porous nanocomposite catalysts subsequently obtained show a high surface area and large mesopores. The Co@N-SiC materials are active, robust, selective and reusable in the hydrogenation of nitroarenes to anilines. Selective hydrogenation of the nitro group in the presence of functional groups sensitive towards hydrogenation, such as the C-C double bond, carbonyl moiety, iodo and nitrile groups, was achieved successfully. We expect that the catalyst synthesis protocol introduced here can be used for the synthesis of a variety of highly active and reusable base metal catalysts.

## Methods

### Synthesis of Co@N-SiC

OH-terminated polyethylene (Mn = 1194 g/mol, Mw = 1506 g/mol, PDI = 1.26) was first dissolved in pyridine followed by the addition of PCS in the weight ratio of 30/70 (PE-OH:PCS). Dehydrocoupling of the Si-H and O-H groups leads to the formation of a block copolymer (PCS-*b*-PE) in which both organic (PE) and inorganic (PCS) blocks are covalentally bonded. As both blocks are immiscible and covalentally bonded, microphase separation leads to the formation of a nanostructured material. Afterwards cobalt complex [Co(phen)_2_](OAc)_2_ was added to achieve a metal to silicon ratio of 1 to 20 (3 wt% Co). The cross-linking of PCS block was started by the addition of dicumyl peroxide (5 wt% with respect to PCS). Preceramic cross-linked material is called a “greenbody”.

Green body was pyrolysed at 750 °C with a heating rate of 1 K/min up to 300 °C and afterwards 5 K/min up to final temperature. The material was held for two hours each at 300, 400 and 500 °C.

### Catalysis

In a typical catalysis run, nitroarene (0.5 mmol), solvent (4 mL) and catalyst (0.5–2 mol%) were charged in a Parr autoclave and reactor was pressurized with hydrogen (4.0 MPa). The autoclave was heated with stirring to a specified temperature and cooled to room temperature at the end of the reaction. The catalyst was separated by centrifugation and products were extracted in diethyl ether and quantified using GC.

## Electronic supplementary material

Supplementary information
