# Supplementary material for: Nitrogen-doped mesoporous SiC materials with catalytically active cobalt nanoparticles for the efficient and selective hydrogenation of nitroarenes
Source: Sci Rep. 2018 Feb 7;8:2567. doi: 10.1038/s41598-018-20976-z (PMC5803264; doi:10.1038/s41598-018-20976-z)
Supplement: Supplementary file 1 — Supplementary information [file 41598_2018_20976_MOESM1_ESM.docx]

**Supporting Information**

Nitrogen-doped mesoporous SiC materials with catalytically active cobalt nanoparticles for the efficient and selective hydrogenation of nitroarenes

Mirco Eckardt^a^, Muhammad Zaheer^b^*, Rhett Kempe^a^*

**Experimental**

General remarks. All reactions were carried out under dry argon using standard Schlenk and glove box techniques. Solvents were dried and distilled from sodium benzophenone before use.

Polycarbosilane (SMP-10) was purchased from Starfire Systems, New York, USA. Phenanthroline and cobalt acetate were purchased from Sigma Aldrich while OH-terminated polyethylene (Mn = 1194 g / mol, Mw = 1506 g / mol, PDI = 1.26) was synthesized in our group via molecular catalysis.

**Characterization**

**BET**. The specific surface area measurements were carried out on Quantachrome (NOVA 2000 e) surface area and pore size analyzer. The pore width and average pore volume was calculated using nonlocal density functional theory (NLDFT, adsorption branch) and silica kernel (cylindrical pore geometry) was applied.

**PXRD**. All X-ray powder diffractograms were recorded by using a STOE STADI-P-diffractometer (CuKα radiation) in θ-2θ -geometry and with a position sensitive detector. All powder samples were introduced into glass capillaries (ø = 0.7 mm, Mark-tubes Hilgenberg No. 10) and sealed prior to the measurements.

**Thermal analysis**. (TGA/DTA) was performed over Thermowaage L81 (Linseis Germany).

**TEM.** Transmission electron microscopy (TEM) was carried out by using a Varian LEO 9220 (200 kV) and a JOEL JEM-2200FS instrument. The sample was suspended in chloroform and sonicated for 5 min. Subsequently a drop of the suspended sample was placed on a grid (Plano S 166–3) and allowed to dry.

**GC**. Gas chromatography (GC) analyses were performed by using an Agilent 6890N gas chromatograph equipped with a flame ionization detector (FID) and an Agilent 19091 J-413 FS capillary column using dodecane as internal standard.


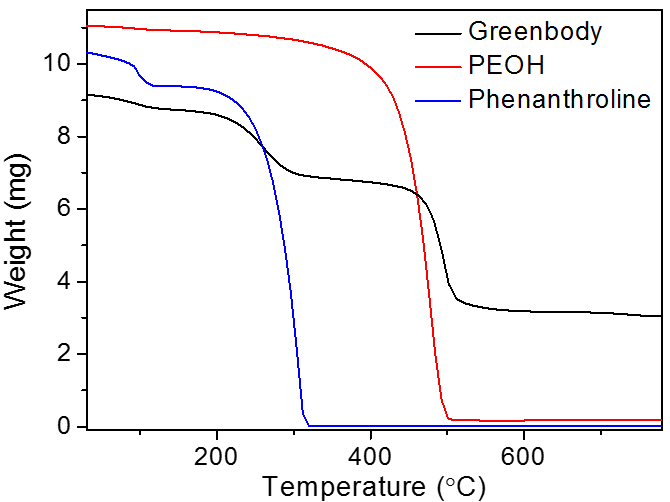


Figure S1: TGA of ligand (phenanthroline) in comparison to PEOH and cross-linked PCS-b-PE (green body).


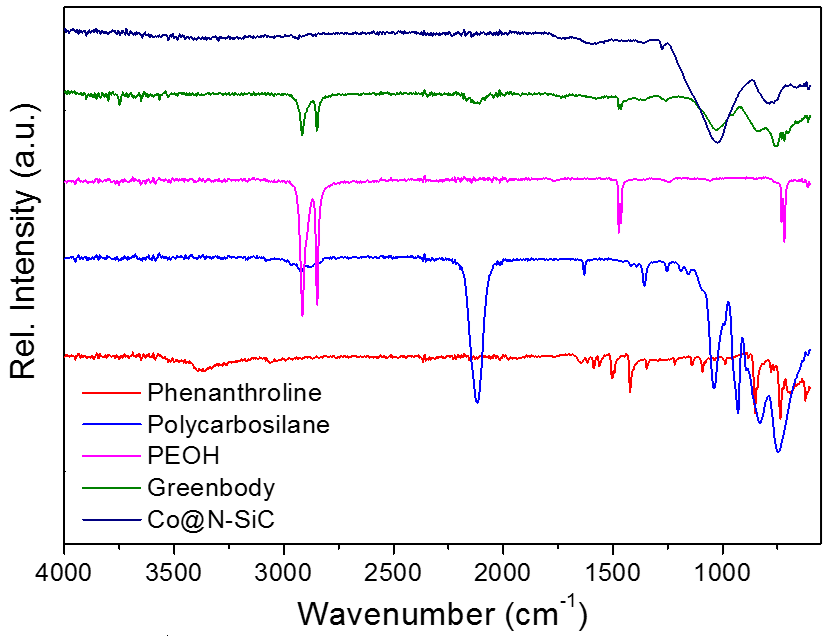


Figure S2: FT-IR spectrum of ligand, ceramic polymer, organic block, cross-linked polymer (green body) and final ceramic material (Co@N-SiC)

Table T1. Effect of solvents on catalytic hydrogenation of nitrobenzene with Co@N-SiC catalyst ^[a]^.

| Entry | Solvent | Yield (%) |
| --- | --- | --- |
| 1 | Toluene | 32 |
| 2 | TEA | 31 |
| 3 | THF | 17 |
| 4 | THF/H2O | 29 |
| 5 | EtOH | 41 |
| 6 | EtOH/H2O | 85 |
| 7 | H2O | 87 |
|  |  |  |

^[a]^ reaction conditions: 0.5 mmol nitrobenzene, 90 ° C., 4.0 MPa H_2_, 0.5 mol% catalyst (0.15 mg Co, 0.0025 mmol, 6.5 Mg, 1), 2 ml of H2O, 2 ml of EtOH, 20 h, yields were determined by means of GC with n-dodecane as the internal standard.

Table T2: Effect of various ligand on surface area and catalytic activity of Co@N-SiC catalysts

|    L1 L2 L3 | | | | |
| --- | --- | --- | --- | --- |
| Entry | Co-complex | S_BET_  (m^2^/g)^a^ | Pore size (nm)^b^ | Yield (%)^c^ |
| 1 | Co(L1)_2_(ac)_2_ | 450 | 2.5-7.5 | 0 |
| 2 | Co(L2) _2_(ac)_2_ | 470 | 2.5-7.5 | 10 |
| 3 | Co(L3) _2_(ac)_2_ | 400 | 2.5-12.5 | >99 |
| 4 | VO(L3) _2_(ac)_2_ | 290 | 2.5-7.5 | 0 |
| 5 | Co(ac)_2_ | 570 | 2.5-7.5 | 0 |

^a^Specific surface area as determined by BET model. ^b^Pore size distribution of various M@N-SiC catalysts using NLDFT (equilibrium mode using carbon kernel with slit /cylindrical pore).

^c^ Yield of aniline obtained in the hydrogenation of nitrobenzene. Reaction conditions: 0.5 mmol nitrobenzene, 90 ° C., 4.0 MPa H_2_, 1 mol% catalyst (0.3 mg Co, 0.005 mmol, 11 mg), 2 ml H_2_O, 2 ml EtOH, 20 h.

VO: Vanadium oxide; ac: acetate; Ph: phenyl
